# Genet dynamics of a regenerating dwarf bamboo population across heterogeneous light environments in a temperate forest understorey

**DOI:** 10.1002/ece3.3793

**Published:** 2018-01-08

**Authors:** Ayumi Matsuo, Hiroshi Tomimatsu, Yushin Sangetsu, Yoshihisa Suyama, Akifumi Makita

**Affiliations:** ^1^ Faculty of Bioresource Sciences Akita Prefectural University Akita Japan; ^2^ Faculty of Science Yamagata University Yamagata Japan; ^3^ Field Science Center Graduate School of Agricultural Science Tohoku University Osaki Miyagi Japan

**Keywords:** bamboo die‐off, canopy gaps, clonal plants, environmental heterogeneity, genotypes, population dynamics, *Sasa kurilensis*

## Abstract

Despite the advantage of plant clonality in patchy environments, studies focusing on genet demography in relation to spatially heterogeneous environments remain scarce. Regeneration of bamboos in forest understoreys after synchronous die‐off provides an opportunity for assessing how they come to proliferate across heterogeneous light environments. In a Japanese forest, we examined genet demography of a population of *Sasa kurilensis* over a 7‐year period starting 10 years after die‐off, shortly after which some genets began spreading horizontally by rhizomes. The aboveground biomass was estimated, and genets were discriminated in 9‐m^2^ plots placed under both canopy gaps and closed canopies. Overall, the results suggest that the survival and spread of more productive genets and the spatial expansion of genets into closed canopies underlie the proliferation of *S. kurilensis*. Compared to canopy gaps, the recovery rate of biomass was much slower under closed canopies for the first 10 years after the die‐off, but became accelerated during the next 7 years. Genet survival was greater for more productive genets (with greater initial number of culms), and the spaces occupied by genets that died were often colonized afterward by clonal growth of surviving genets. The number of genets decreased under canopy gaps due to greater mortality, but increased under closed canopies where greater number of genets colonized clonally from outside the plots than genets died. The colonizing genets were more productive (having larger culms) than those originally germinated within the plots, and the contribution of colonizing genets to the biomass was greater under closed canopies. Our study emphasizes the importance of investigating genet dynamics over relevant spatiotemporal scales to reveal processes underlying the success of clonal plants in heterogeneous habitats.

## 1 INTRODUCTION

Many plants (c. 70% in the temperate zone) possess the capacity to reproduce vegetatively via rhizomes and stolons (Klimeš, Klimešova, Hendriks, & van Groenendael, 1997). Such clonal plants, including many invasive exotics (Canavan et al., 2016; Liu et al., 2006; Pyšek, 1997), often dominate terrestrial habitats, such as grasslands, salt marshes, and understoreys of temperate and boreal forests. Clonal plants also appear to increase its abundance in response to global change drivers such as habitat fragmentation (Tomimatsu et al., 2011) and nutrient deposition (Dickson, Mittelbach, Reynolds, & Gross, 2014; Gough et al., 2012), with important consequences for community structure and function.

The widespread success and dominance of clonal plants may at least partly be due to their ability of genets (i.e., a genetic individual arising from seed) to forage for resources such as light, water, and nutrients in patchy environments in terms of resource supply and to share acquired resources among connected ramets (i.e., individual shoots) (Alpert & Stuefer, 1997; Hutchings & Wijesinghe, 1997; de Kroon & Hutchings, 1995). Foraging and clonal integration can alleviate local deficiency of resources and therefore benefit whole genets in heterogeneous habitats (Amsberry, Baker, Ewanchuk, & Bertness, 2000; Pennings & Callaway, 2000; Roiloa, Alpert, Tharayil, Hancock, & Bhowmik, 2007; Saitoh, Seiwa, & Nishiwaki, 2002). Some species have even been shown to grow more vigorously when the same quantity of resources is supplied heterogeneously than homogeneously (Birch & Hutchings, 1994; Day, John, & Hutchings, 2003; He, Alpert, Yu, Zhang, & Dong, 2011; Song et al., 2013; Wang, Lei, Li, & Yu, 2012). As individual genets expand horizontally by clonal growth, intraspecific competition is expected to become stronger through time (Abbott & Stachowicz, 2016; Silvertown, 2008; Watkinson & Powell, 1993). Theoretically, competition among neighboring genets can further contribute to their proliferation, because the spread and dominance of successful genotypes with high growth rates enhance the productivity of populations (Drummond & Vellend, 2012; Loreau & Hector, 2001; Tomimatsu, Nakano, Yamamoto, & Suyama, 2014). In particular, proliferation in resource‐poor microsites may depend on the dominance of large genets that spread widely and take advantage of more resource‐rich environments, whereby parent plants can provide resources to new shoots under resource‐poor environments. Even though both of these processes, vegetative expansion and competitive interactions, occur at the genet level, studies of genet dynamics in clonal plants have long been deterred by the difficulty of discriminating distinct genets and observing over relevant spatiotemporal scales (Arnaud‐Haond, Duarte, Alberto, & Serrão, 2007; Eriksson, 1993).

Early attempts to study genet dynamics of natural populations have particularly focused on recruitment strategies and emphasized the role of disturbances for successful seedling recruitment (Barrett & Silander, 1992; Eriksson & Bremer, 1993; Hartnett & Bazzaz, 1985; de Steven, 1989). Subsequent work using molecular markers has supported this view by showing that genotypic diversity of populations tended to decrease with time elapsed since the last disturbance (Lambertini, Gustafsson, Frydenberg, Speranza, & Brix, 2008; Scheepens, Veeneklaas, van de Zande, & Bakker, 2007; Silvertown, 2008; Travis & Hester, 2005; but see Reusch, 2006; Verburg, Mass, & During, 2000). Although the prevalence of clonality in older populations infers competitive dynamics and spatial expansion of successful genets, many studies measured genotypic diversity only at a single point in time to infer such dynamics (but see Becheler, Benkara, Moalic, Hily, & Arnaud‐Haond, 2014). Moreover, despite the advantage of clonality in patchy environments, only a limited number of studies have focused on the dynamics in habitats with explicit patterns of environmental heterogeneity (Eriksson & Bremer, 1993; Kudoh, Shibaike, Takasu, Whigham, & Kawano, 1999; de Steven, 1989; Vandepitte, Roldán‐Ruiz, Leus, Jacquemyn, & Honnay, 2009). More detailed examinations of genet demography in relation to spatially heterogeneous environments are more laborious, but necessary to understand the processes responsible for the success of clonal species.

**Figure 1:**
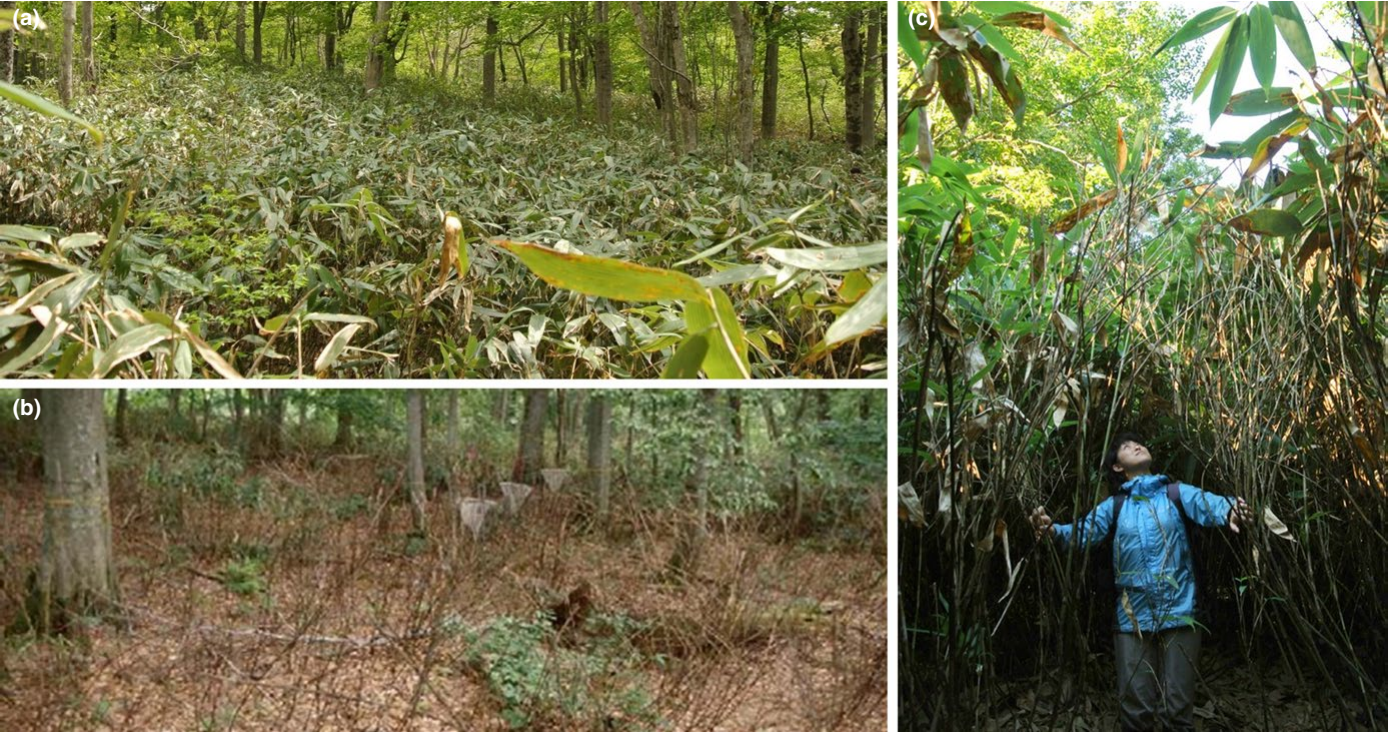
(a) Dense forest understorey dominated by *Sasa kurilenesis*. (b) Extensive dieback followed the synchronous flowering in 1995. (c) Culms grow over 3 m height in canopy gaps. Photo credit: (a) H. Tomimatsu, (b) A. Makita, and (c) H. Tomimatsu

Here, we investigated long‐term population dynamics of the dwarf bamboo, *Sasa kurilensis* (Rupr.) Makino et Shibata (Poaceae), in an old‐growth Japanese beech forest. *Sasa kurilensis* is a highly clonal plant that propagates vegetatively with persistent underground rhizomes and dominates understoreys of temperate deciduous forests (Figure 1a). The dense bamboo understoreys have long been thought to interfere with regeneration of canopy trees and therefore are key components in temperate forests of East Asia and South America (Abe, Miguchi, & Nakashizuka, 2001; Caccia, Kitzberger, & Chaneton, 2015; Giordano, Sánchez, & Austin, 2009; Nakashizuka, 1988; Taylor & Zisheng, 1992). Like many other bamboos (Campbell, 1985; Janzen, 1976; Ueda, 1961), *Sasa* species are typically monocarpic and undergo gregarious flowering followed by mass seeding and die‐off although the exact flowering interval is not known. In 1995, mass flowering and die‐off took place across >1,000 ha in Akita, northern Japan (Makita, Makita, & Nishiwaki, 1995; Figure 1b). The process of population recovery following the die‐off provides an opportunity for assessing how *S. kurilensis* comes to proliferate across the forest understorey. Some areas of the site did not undergo flowering and die‐off, which created a mosaic of die‐off (dead) and nonflowered (live) bamboo patches (Makita et al., 1995). Our observations indicate that the initial recovery of biomass in the die‐off patches was greatly influenced by the light environment, such that the recovery was much slower under closed canopies compared to canopy gaps (Aikawa, Tomimatsu, Matsuo, Sangetsu, & Makita, 2017). In nonflowered (live) patches, however, biomass density (aboveground biomass per unit area) did not depend on light conditions, suggesting that *S. kurilensis* will eventually develop dense thickets across the whole site regardless of light conditions. Given that genets of dwarf bamboos can potentially spread over large areas across a broad range of light conditions (Matsuo, Suyama, Sangetsu, Fuji, & Makita, 2008; Saitoh, Seiwa, Nishiwaki, Kanno, & Akasaka, 2000) and that clonal integration was suggested as important in the persistence under closed canopies (Saitoh et al., 2002), clonal expansion from higher‐light into shaded microsites may play a crucial role in the proliferation of *S. kurilensis* across the forest understorey.

Using 9‐m^2^ plots placed under both canopy‐gap and closed‐canopy microsites in a die‐off patch, we examined biomass recovery and genet demography over a 7‐year period starting 10 years after the die‐off, shortly after which some genets initiated to spread horizontally by rhizomes (Kudoh & Ujiie, 1990; Makita, 1992). In particular, we focused on testing four specific predictions of the hypothesis that competitive interactions and spatial expansion toward closed‐canopy microsites underlie the development of dense thickets across spatially heterogeneous light environments. (1) Genet mortality should be density dependent, with greater mortality in canopy gaps where initial genet density is greater due to higher levels of seedling recruitment (Makita, Abe, Miguchi, & Nakashizuka, 2004). (2) Genet survival should be size dependent, with more productive genets being more likely to survive and spread laterally. (3) A proportionately greater number of genets should spread clonally into closed‐canopy plots compared to canopy‐gap plots, although the *absolute* number of genets that spread clonally into plots may be greater under canopy gaps due to higher genet densities. (4) Genets that spread clonally into closed‐canopy plots should, on average, be more productive than those that originally germinated and persist under closed canopies. Although we employed only six plots because of an extensive effort necessary to distinguish genets, we also attempted to compare the contribution of clonal expansion to biomass recovery, which should be greater under closed canopies.

## 2 MATERIALS AND METHODS

### 2.1 Study species and site

*Sasa kurilensis* is an evergreen dwarf bamboo distributed in Japan, Korea, Sakhalin, and Kuril Islands. The species grows up to ~3 m height (Figure 1c), is one of the tallest in the genus, and is dominant in the areas of heavy snowfall in winter (e.g., Hokkaido and the Sea of Japan side of Honshu Island). This may be because *S. kurilensis* produces winter buds higher on culms, which are protected from deeper snow, than other congeners and thus is able to develop culm branches from these buds (Suzuki, 1978). While McClure (1966) recognized two basic rhizome types in bamboos, pachymorph (short and thick) and leptomorph (long and creeping), *S. kurilensis* propagates vegetatively through a mixture of both rhizome types (Makita, 1998). Specifically, each seedling initially develops a clump with pachymorph rhizomes for at least 6 years (often >10 years; Kudoh & Ujiie, 1990; Makita, 1992), only after which it begins to extend via leptomorphic rhizomes that connect the original clump with the new clumps. Although the mating system of *S. kurilensis* has not been examined, *Sasa* species are generally self‐compatible and often show high selfing rates (e.g., Matsuo et al., 2014; Mizuki et al., 2014).

We conducted the study in an old‐growth forest located near Lake Towada, Akita, Japan (40°24′N, 140°52′E; 670 m a.s.l.). Mean annual temperature and rainfall were 6.7°C and 1,666 mm, respectively. The forest is dominated by *Fagus crenata*, and other overstory species include *Magnolia obovata*,* Acer pictum*,* A. japonicum*, and *Tilia japonica*. The understorey light environment was quite heterogeneous over the scale of meters due to many canopy gaps. Canopy openness, quantified using hemispherical photographs taken above the understorey canopy of *S. kurilensis* in August 2005 across 85 points in our study site (~1 ha), varied greatly from 4.2% to 26.0% with a mean of 10.8% (Aikawa et al., 2017). The spatial heterogeneity in light reflects long‐term canopy‐gap dynamics such that canopy gaps may not soon be filled because tree regeneration is severely limited by the dense thickets of *S. kurilensis* and thus may depend on the timings of both bamboo die‐offs and masting events in *F. crenata* (Abe et al., 2001; Nakashizuka, 1988). Other understorey plants were much less frequent and not a major factor affecting the light condition for *S. kurilensis*. The synchronous flowering was followed by mass seeding and extensive dieback in autumn 1995, which resulted in 27.9 ± 7.4 seedlings per m^2^ in canopy gaps and 14.4 ± 9.4 seedlings per m^2^ under closed canopies (mean ± *SD*) in 1996 (Makita et al., 2004). The greater seedling density in canopy gaps was attributable to both greater seed production (Makita et al., 1995) and subsequent establishment (Makita et al., 2004). While *S. kurilensis* flowered sporadically in the live patches, seedling recruitment following sporadic flowering is strongly limited because of low seed set and high predation pressure on seeds (Makita et al., 2004; Mizuki et al., 2014). Because the nonflowered patches have not undergone mass flowering (Makita et al., 2004) and there is no persistent seed bank (Makita, 1992), few seedlings, if any, could have recruited since then. Thus, the development of our population can be characterized by the “initial seedling recruitment” (sensu Eriksson, 1993), with no recruitment after the establishment of the initial cohort in 1996.

### 2.2 Field survey

We started the survey in August 2005 (i.e., 10 years after the die‐off) because genets of *S. kurilensis* develop original clumps often for >10 years before spreading laterally (see Section 2.1). We established six 3 × 3 m plots under three levels of light environment within a die‐off patch: Two were selected under closed forest canopies (“closed canopy”: C‐1, C‐2), two under large canopy gaps (“canopy gap”: G‐1, G‐2), and two under intermediate forest canopies (“intermediate canopy”: I‐1, I‐2). The visually estimated degree of canopy closure corresponded well to the canopy openness quantified with hemispherical photographs (6.1% and 7.4% for closed canopy; 9.7% and 10.8% for intermediate canopy; 16.4% and 15.1% for canopy gap). The canopy openness for closed‐canopy plots falls below the 10th percentile of the openness across the 85 points in our site (see Section 2.1). In addition, canopy openness did not greatly change during the 7 years of study (Figure S1). Although the replicates of die‐off patches would be necessary to confirm the generality of our study, the die‐off patch was quite large (extended far beyond our study area) and intermingled with nonflowered patches, so that we could not even determine whether other die‐off patches exist. The plots were >15 m apart from one another, while the paired plots with the same levels of light environment were placed >50 m apart.

To estimate the aboveground biomass of each plot, we recorded the age and diameter at ground level of all culms observed in the plots both in August 2005 (*N *=* *968) and in August 2012 (*N *=* *632). Culm age was classified based on its branching pattern into three classes: 0 (current year), 1 (1 year old), and 2+ (two or more years old). We established the allometric relationship of the aboveground biomass (dry weight) of each culm to its age and diameter by harvesting 65 culms with full range of sizes in the population. Their tissues were dried in an oven at 70°C for at least 48 hr and weighed. We then performed analysis of covariance (ANCOVA) of the biomass of culms (log‐transformed) with culm age as a categorical, fixed effect and the diameter at ground level (log‐transformed) as a covariate. The nonsignificant interaction term (*p *=* *.57) was removed to obtain the final model (*R* ^2^ = .97), such that: log_10_ [aboveground biomass (g)] = −1.12 + 2.52 log_10_ [diameter at ground level (mm)] + *x*, where *x* is 0 for age 0, 0.21 for age 1, and 0.33 for age 2+ (Figure S2). We calculated the aboveground biomass of each plot by summing the biomass of all culms estimated with the allometric relationship.

To investigate genet demography, we discriminated genets using both field survey and DNA analysis. In 2005, we first traced the extending patterns of rhizomes by excavating the top ~5 cm of soil using spoons. We started each excavation at a culm in a plot and then uncovered all rhizomes and associated culms connected to that culm until the entire clonal fragment within the plot was revealed. We then collected small leaf samples from each clonal fragment for DNA analysis (*N *=* *196) to identify genets of all the culms in the plots (*N *=* *968). We also identified genets that spread clonally from outside the excavated plots based on the extending directions of rhizomes (Figure S3). The excavation was performed carefully not to disturb roots, and the soil was returned to the field on the day of excavation. In 2012, we collected leaf samples from all the culms for genet identification by DNA analysis (*N *=* *632), because no culms that were observed in the 2005 survey persisted until the 2012 survey.

### 2.3 Microsatellite genotyping and genet identification

We extracted total DNA from ~2 mg of freeze‐dried leaf tissues using a modified cetyltrimethyl ammonium bromide (CTAB) protocol (Murray & Thompson, 1980). All the samples were genotyped with seven polymorphic microsatellite loci: *Sasa223*,* Sasa500*,* Sasa718*,* Sasa946* (Kitamura, Saitoh, Matsuo, & Suyama, 2009), *BWSS‐4*,* BWSS‐7* (Miyazaki, Ohnishi, Hirayama, & Nagata, 2009), and *ST57* (Table S1). Multiplex polymerase chain reactions (PCRs) were performed using a GeneAmp PCR System 9700 (Applied Biosystems, Foster City, CA, USA) as described by Matsuo et al. (2014). The PCR products were electrophoresed on an ABI PRISM 3130xl DNA Analyzer (Applied Biosystems), and the resulting data were analyzed with GeneMapper 4.0 (Applied Biosystems). Genets were discriminated based on multilocus genotypes (MLGs) of microsatellite loci. MLGsim 2.0 (Stenberg, Lundmark, & Saura, 2003) was used to calculate the probability (*p*_sex_) that repeated MLGs arose through independent sexual reproduction, taking into account departures from Hardy–Weinberg equilibrium (Arnaud‐Haond et al., 2007). The probabilities were ≤2.80 × 10^−3^ for all MLGs, so that we could reasonably assume that any repeated MLGs arose through clonal growth. When MLGs differed only by two base pairs (i.e., one microsatellite motif) in one allele at one locus but shared the same alleles at all the other loci, we considered that the variation between them was derived from somatic mutations and grouped them into a single genet.

### 2.4 Data analysis

To test whether the change in the number of genets over the 7 years differed among the three light levels, we used a χ^2^ contingency test because of only two plot replicates for each light level. The two replicates were combined to produce a 2 × 3 table.

To analyze genet survival rate from 2005 to 2012, we used a generalized linear mixed model (GLMM), which included light condition (closed canopy, intermediate canopy, and canopy gap) and three attributes of genets (heterozygosity, the number of culms, and the average diameter of culms, explained in more detail below) as fixed factors, and plot as a random factor, assuming a binomial error distribution and a logit‐link function. In this analysis, the genets that were found in 2005 but disappeared in 2012 were considered as “dead” rather than escaped from the plots by moving their entire location. Although we have no conclusive evidence that death occurred, most disappeared genets were likely to have died because genets of *S. kurilensis* tend not to begin spreading laterally for >10 years after germination (Kudoh & Ujiie, 1990; Makita, 1992). In addition, genets with slow growth rates did not spread even after 20 years (E. Kudo, A. Matsuo, H. Tomimatsu & A. Makita, 2015). Although some disappeared genets that initially occurred only on the periphery of the plots might have survived outside the plots, excluding such genets (found only within 50 cm from plot edges) did not affect the interpretation of our data. As the attributes of genets, we employed heterozygosity (the number of heterozygous microsatellite loci), the number of culms (ln‐transformed), and the average diameter of culms, all of which were obtained in the 2005 survey. We used the latter two as complementary indices of genet size. While both the number and average diameter of culms are expected to increase as genets grow, the number of culms in our plots may not well reflect genet biomass if a plot covers only a part of the whole genet. In addition, we did not measure the length (and thus height) of culms because the length of individual culms strongly correlated with its diameter (Pearson's *r *=* *.94; *N *=* *59, *t *=* *21.9, *p *<* *.001). We obtained the final model by removing nonsignificant interaction terms in a stepwise manner as long as Akaike information criterion (AIC) declined. To estimate the degree to which the surviving genets spread horizontally, the spatial extent of individual genets (maximum linear distance between culms belonging to the focal genet) was calculated for each plot both in 2005 and in 2012.

Although our study did not directly examine how the attributes of genets relate to the success of clonal expansion (we only assessed genet dynamics within the plots and did not have precise data of spatial genet dynamics over a larger area), we assessed whether the genets that colonized the plots by extending rhizomes after establishing elsewhere (“colonizing genets”) differed in their number and attributes from those that have originally germinated and persisted within the plots (“original genets”). That is, the colonizing genets include not only those that were not detected in 2005 but appeared in 2012 (because no seedlings recruited during this period) but also those that have colonized the plots before the 2005 survey, which was revealed by the excavation of soil (see Section 2.2). A parallel study indicates that while a single colonizing genet (#108 for Plot G‐2) originated from the adjacent live patch, the contribution of this nonflowered genet to biomass recovery in the plot was quite limited (1.8%; Ohya, Y., Tomimatsu, H. & Makita, A., unpublished data).

The analysis of colonizing genets was performed in three ways using the 2012 data. First, to test whether the proportion of colonizing genets differed among the light levels, we used a χ^2^ test on a 2 × 3 contingency table as described earlier. Second, to test whether colonizing and original genets differed in their attributes, we employed GLMMs and a linear mixed model (LMM) analyses. GLMMs were used to analyze heterozygosity (with a Poisson error distribution and a log‐link function) and the number of culms (with a negative binomial error distribution and a log‐link function), while a LMM was used to analyze the average diameter of culms. These models included the origin of genets (i.e., colonizing vs. original) and light condition as fixed factors and plot as a random factor, and again, we removed nonsignificant interaction terms based on their AIC. In these analyses, we particularly focused on the average diameter of culms as an index of genet size, because it likely better reflects the size of colonizing genets that spread vegetatively from outside the plots. Finally, to assess the contribution of clonal expansion to biomass recovery, we compared the proportion of colonizing genets to the total aboveground biomass among plots. We evaluated the data qualitatively, however, as the small sample size (i.e., six plots) precluded statistical comparisons between the light levels. We did not perform this analysis for the 2005 survey, because only three colonizing genets were detected.

All statistical analyses were performed using R 3.1.2. The GLMM and LMM analyses were performed using the *glmer* and *lmer* functions of the “lme4” package (Bates et al., 2014) of R, respectively. In the GLMM analyses, we used Wald *Z* tests to assess the statistical significance of fixed terms because we found no evidence of overdispersion (Bolker et al., 2008).

## 3 RESULTS

### 3.1 Changes in culm density and aboveground biomass

**Figure 2:**
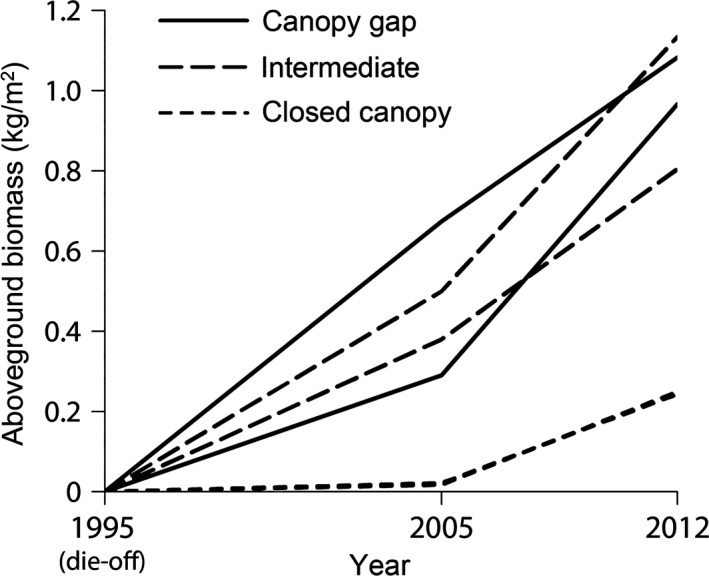
Change in aboveground biomass of *Sasa kurilensis* for six 9‐m^2^ plots since the die‐off in 1995. For convenience, the aboveground biomass in 1995 was set to zero although some dead culms were still there. The two lines for the plots under closed canopies are hard to be distinguished because they are almost identical

Over the 7 years, culm density increased under closed canopies but decreased under canopy gaps and intermediate forest canopies (Table 1). In contrast, mean biomass of individual culms increased less markedly under closed canopies. As a result, although the aboveground biomass showed no clear difference between plots under canopy gaps and intermediate canopies both in 2005 and in 2012, the biomass in these plots was much greater than those under closed canopies (Figure 2), consistent with our observation that biomass recovery was much slower under closed canopies. Compared to the first 10 years after the die‐off (i.e., 1995–2005), the rate of biomass recovery under closed canopies appears to have accelerated since 2005: Aboveground biomass increased more than 11‐fold over the 7 years of study (C‐1: 0.021 kg/m^2^ in 2005, 0.243 kg/m^2^ in 2012; C‐2: 0.017 kg/m^2^ in 2005, 0.249 kg/m^2^ in 2012).

**Table 1:** Density and size of culms and genets of *Sasa kurilensis* for six 9‐m^2^ plots under three levels of light conditions

Light condition	Plot	Number of culms		Mean estimated biomass of culms (g)		Number of genets		Number of genets in 2012 classified by demography		Genet mortality 2005–2012 (%)	Average spatial extent of genets (m)		Maximum spatial extent of genets (m)	
		2005	2012	2005	2012	2005	2012	Original	Colonizing		2005	2012	2005	2012
Closed canopy	C‐1	55	58	3.5	37.8	15	24	12	12	20.0	0.13	0.25	2.43	3.50
	C‐2	70	87	2.2	25.7	17	21	11	10	35.3	0.13	0.63	0.43	3.11
Intermediate canopy	I‐1	216	128	12.1	67.9	47	44	29	15	38.3	0.35	0.58	2.20	2.46
	I‐2	227	134	19.8	76.1	76	48	29	19	61.8	0.41	0.79	2.54	3.37
Canopy gap	G‐1	194	106	31.3	91.9	45	38	23	15	48.9	0.40	0.71	2.57	3.14
	G‐2	206	119	16.6	60.8	101	42	17	25	83.2	0.43	0.40	3.00	3.67

Note that average and maximum spatial extents of genets were evaluated only within the plots, although many genets spread over larger areas in 2012.

### 3.2 Change in the number of genets

Over the 7 years, the number of genets changed differently among the three light levels (*df* = 2, χ^2^ = 15.6, *p *<* *.001). The number of genets decreased by 6.4%–58.4% under canopy gaps and intermediate canopies with high culm and genet densities (Table 1). Conversely, under closed canopies with low initial densities, the number of genets increased by 64.3% and 23.5% in the C‐1 and C‐2 plots, respectively. While the proportion of colonizing genets did not differ among the light levels in 2012 (*df* = 2, χ^2^ = 3.5, *p *=* *.18), the mortality was lower under closed canopies (Table 1; see Section 3.3 for statistics), where, as a result, a greater number of genets colonized the plots than died. Note that the total number of colonizing genets was greater in canopy gaps.

### 3.3 Genet survival and spread

**Figure 3:**
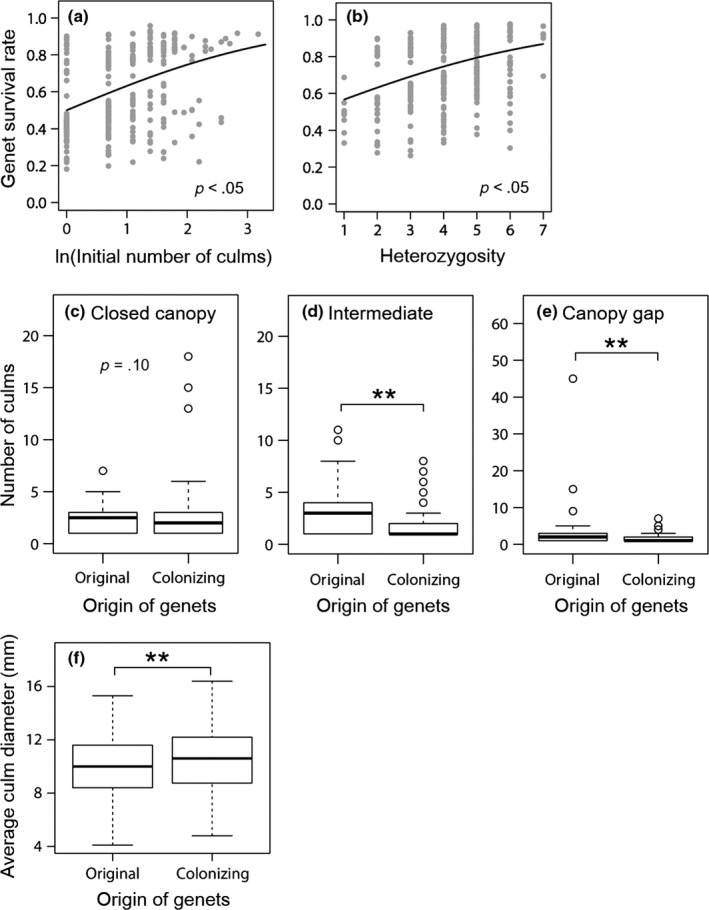
Results from analyses of genet dynamics based on the GLMMs and LMM (Table 2). Partial residual plots showing the effects of (a) the initial number of culms and (b) heterozygosity (the number of heterozygous loci) on genet survival rate during 2005–2012. The fitted lines are from the final model (see Table 2a for estimated parameters). (c–e) A number of culms for original vs. colonizing genets in 2012 are separately shown for three levels of light condition because of the significant origin × light interaction (Table 2c). (f) Average diameter of culms for original versus colonizing genets in 2012. Asterisks denote significant differences between original versus colonizing genets (*p *<* *.01)

The GLMM analysis showed that, compared to closed canopies, genet survival rate was significantly lower (and thus mortality rate was greater) under canopy gaps (*Z *= −3.45, *p *<* *.001) and intermediate canopies (*Z *= −4.00, *p *<* *.001; Table 2a). The GLMM analysis also indicated that the survival of genets increased with its heterozygosity (*Z *=* *2.14, *p *<* *.05) and initial number of culms (*Z *=* *2.12, *p *<* *.05; Table 2a; Figure 3a,b). While the interaction term between the average diameter of culms and light condition was also significant, the results suggest greater rates of survival for more productive genets with greater initial number of culms. Both the average and maximum spatial extents of surviving genets tended to increase over the 7 years (Table 1) even though these were underestimated in 2012 as many genets spread extending outside the plots, indicating that many surviving genets spread horizontally. The spaces previously occupied by dead genets were often colonized afterward by clonal growth of other genets (Figure S4).

**Table 2:** (a) Effects of genet traits (heterozygosity, initial number of culms, and average diameter of culms), light condition, and their interactions on genet survival rate during 2005–2012 and (b–d) the effects of genet origin (original vs. colonizing), light condition, and their interactions on the three genet traits in 2012, analyzed with either GLMMs or LMM

	Estimate	*SE*	*Z* or *t*	*p*
(a) Genet survival rate during 2005–2012, analyzed with GLMM				
Intercept	−0.71	1.09	−0.65	.51
Heterozygosity	0.27	0.12	2.14	**<.05**
Number of culms	0.52	0.24	2.12	**<.05**
Average diameter of culms	0.04	0.25	0.19	.85
Light				
Intermediate canopy	−5.45	1.36	−4.00	**<.001**
Canopy gap	−4.35	1.26	−3.45	**<.001**
Interactions				
Average diameter × intermediate canopy	0.76	0.30	2.52	**<.05**
Average diameter × canopy gap	0.35	0.27	1.27	.20
(b) Heterozygosity in 2012, analyzed with GLMM				
Intercept	1.42	0.08	17.62	**<.001**
Origin (colonizing)	−0.07	0.06	−1.07	.28
Light				
Intermediate canopy	0.00	0.09	0.00	.99
Canopy gap	0.06	0.09	0.70	.48
(c) Number of culms in 2012, analyzed with GLMM				
Intercept	0.99	0.18	5.46	**<.001**
Origin (colonizing)	0.34	0.24	1.39	.16
Light				
Intermediate canopy	0.20	0.21	0.94	.34
Canopy gap	0.35	0.22	1.60	.10
Interactions				
Colonizing × intermediate canopy	−0.80	0.31	−2.54	**<.05**
Colonizing × canopy gap	−1.02	0.31	−3.23	**<.01**
(d) Average diameter of culms in 2012, analyzed with LMM				
Intercept	7.65	0.82	9.36	**<.01**
Origin (colonizing)	0.93	0.28	3.34	**<.01**
Light				
Intermediate canopy	2.64	1.13	2.33	.09
Canopy gap	2.96	1.13	2.61	.07

Nonsignificant interaction terms were dropped using AIC to obtain final models. We used *Z* and *t* statistics for GLMM and LMM analyses, respectively.

Bold values indicate statistically significant terms (*p *<* *.05).

### 3.4 Colonizing versus original genets

In 2012, the average diameter of culms was significantly greater for colonizing versus original genets (*t *=* *3.34, *p *<* *.01; Table 2d; Figure 3f), suggesting that the colonizing genets were generally more productive regardless of light conditions. The number of culms of colonizing genets was considerably lower than that of original genets under canopy gaps (*Z *= −2.86, *p *<* *.01) and intermediate canopies (*Z *= −2.82, *p *<* *.01; Table 2c; Figure 3d,e), indicating that colonizing genets were less abundant in these plots. In contrast, under closed canopies, there was a weak tendency for colonizing genets to be more abundant (*Z *=* *1.61, *p *=* *.10; Figure 3c). In particular, three colonizing genets (outliers in Figure 3c; #010 for C‐1, #002 and #008 for C‐2 in Figure S4) were far more abundant than the other genets.

**Figure 4:**
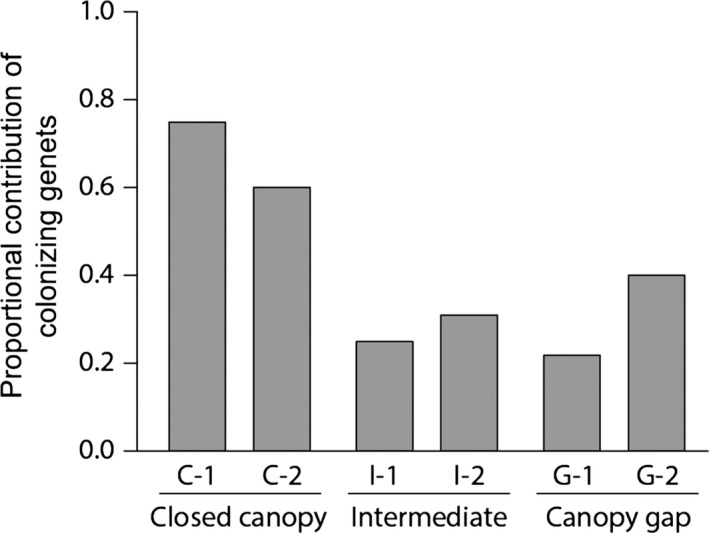
Contribution of clonal growth to the aboveground biomass of *Sasa kurilensis*, quantified as the proportional contributions of colonizing genets, for six 9‐m^2^ plots in 2012

The proportional contributions of colonizing genets to the total aboveground biomass were greater under closed canopies (C1: 73.1% and C2: 59.8%) compared to canopy gaps and intermediate canopies (≤40.7%; Figure 4), although the small number of plots precluded statistical comparisons between the light levels. Under closed canopies, the three colonizing genets described earlier contributed as much as 44.7% and 30.7% to the total biomass of the C‐1 and C‐2 plots, respectively.

## 4 DISCUSSION

Competitive interactions and clonal expansion into less favorable habitats have been implicated as potential mechanisms that may promote the proliferation of clonal species (Amsberry et al., 2000; Pennings & Callaway, 2000; Saitoh et al., 2002; Stuefer et al., 2009; Tomimatsu et al., 2014). However, genet demography has rarely been investigated in relation to spatially heterogeneous environments. Our study analyzed genet demography and biomass recovery of *S. kurilensis* following the synchronous flowering and subsequent die‐off to examine whether such processes underlie the development of dense thickets across heterogeneous light environments, although the extensive effort necessary to conduct the research set limits to the number of plots.

The mortality rates of genets, which could have been slightly overestimated due to the difficulty of detecting death, appeared to be density dependent, with greater mortality under canopy gaps and intermediate canopies with greater initial densities (Tables 1 and 2a). Moreover, spaces previously occupied by genets that died after the 2005 survey were often colonized by other genets in 2012 (Figure S4). These results emphasize competitive interactions among neighboring genets, although the effect of individual heterozygosity on survival (Figure 3b) may indicate that some mortality was attributable to inbreeding depression which is caused by the genome‐wide increase in homozygosity due to inbreeding (Milller & Coltman, 2014). While our two indices of genet size, the number and average diameter of culms, were positively but weakly correlated (*r *=* *.28), the number of culms was a better predictor of genet survival (Figure 3a; Table 2a). This may be because most genets still consisted of only a few culms in 2005 (mean, 3.2), and thus, the mortality risk through competition was better spread in genets with more culms. Given that our study area was an even‐aged cohort, the results also suggest that the outcome of competition reflects the fitness differences caused by differential growth rates. A few experimental studies that examined the interaction among genets showed that populations with multiple genets were likely to become dominated by particular genets with high growth rates (Stuefer et al., 2009; Tomimatsu et al., 2014; but see Abbott & Stachowicz, 2016). The survival and spread of more productive genets are expected to increase productivity of populations if such genets replace less productive ones (sensu “selection effect”; Loreau & Hector, 2001) and may contribute to the proliferation of *S. kurilensis*.

Genets that spread vegetatively into plots (“colonizing genets”) produced thicker (and taller) culms than those which originally germinated and persisted there (“original genets”) (Figure 3f). In addition, although the proportion of colonizing to the total number of genets did not differ among the light levels (Table 1), some genets that colonized the closed‐canopy plots became fairly abundant (Figure 3c). In contrast, colonizing genets were still less abundant under canopy gaps and intermediate canopies (Figure 3d,e). These results explain well why the proportional contribution of colonizing genets to the aboveground biomass was greater under closed canopies (Figure 4). Together with the result that the number of genets increased rather than decreased under closed canopies even without seedling recruitment (Table 1), our data suggest that clonal expansion into closed‐canopy microsites was crucial for the accelerated recovery of biomass since 2005 (Figure 2).

While whether spatial expansions of genets into closed‐canopy microsites are common across die‐off patches warrants further study, the colonizing genets in the closed‐canopy plots likely originated from surrounding higher‐light microsites because the canopy openness of these plots was rather low compared to most other areas of our site (see Section 2.2). In fact, a preliminary survey examining the spatial extent of 20 of 45 genets in the closed‐canopy plots by tracing rhizomes indicates that the original genets still remained within or around the plots, whereas many colonizing genets extended rhizomes for >10 m (max. 39 m) from higher‐light microsites toward the closed‐canopy plots (E. Kudo, A. Matsuo, Y. Kaneko, H. Tomimatsu & A. Makita, unpublished data), suggesting that the colonizing genets were larger in size and initially established themselves in more favorable habitats. Experimental studies suggest that the exchange of carbohydrates between ramets is largely driven by source–sink relationships (He et al., 2011; Roiloa et al., 2007; Saitoh et al., 2002; Stuefer, During, & de Kroon, 1994). Thus, the genets that extend over large areas may have translocated carbohydrates between connected ramets from higher‐light (“source”) into closed‐canopy (“sink”) microsites via physiological integration and contributed to the accelerated recovery of biomass. Further validation of our results will also require a longer‐term monitoring of genet demography to test whether colonizing genets continue to increase their abundance and finally replace original, less productive genets under closed canopies.

It should be noted, however, that whereas the average diameter of culms tended to differ between the light levels, colonizing genets produced larger culms not only under closed canopies but also in canopy gaps (Table 2d). Considering that not all culms are able to receive enough light even in canopy gaps due to competition, more productive genets that produced thicker (and thus taller) culms were more likely to exploit disproportionately greater amounts of light and use fixed assimilates to spread to a greater extent. Although the dichotomy of colonizing versus original genets used in this study does not necessarily mean that the original genets did not spread laterally, the colonizing genets in canopy gaps were also, on average, likely to be more productive and spread over a larger area if some original genets still remained within the plots because of low growth rates.

Clonal expansion and the survival of more productive genets predict the development of very large genets with time. In fact, the adjacent nonflowered (live) patches, where a considerable period of time should have passed since the last flowering and die‐off, were dominated by only a few genets that covered a much larger area than those in the die‐off patch (Matsuo et al., 2008; Y. Ohya, H. Tomimatsu & A. Makita, unpublished data). The largest genet was found to cover >3,400 m^2^ (Matsuo et al., 2008). Because it is commonly thought that clonal plants colonize more favorable patches to forage for resources (de Kroon & Hutchings, 1995), it may seem curious that *S. kurilensis* spreads even into closed‐canopy microsites and develops very large genets. This could be explained by three plausible, but not mutually exclusive, factors. First, light conditions in forest understoreys are not temporally constant due to the formation and closure of canopy gaps. Simulation studies suggest that temporal variation in resource availability increases the advantage of clonal integration because interconnected ramets can complement local shortage of resources (Kun & Oborny, 2003; Mágori, Oborny, Dieckmann, & Meszena, 2003). Second, if above‐ and belowground resources are negatively associated such that light availability is higher but nutrient availability is lower in canopy gaps compared to closed canopies, reciprocal translocation of resources between connected ramets could enhance growth of whole genets (Alpert & Stuefer, 1997; Hutchings & Wijesinghe, 1997). However, much greater initial growth under canopy gaps (Figure 2) suggests that canopy gaps are much more favorable habitats for *S. kurilensis*. Finally, previous studies showed that the development of dense understorey of *S. kurilensis* under closed canopies impedes the establishment of seedling banks of beech trees by indirect effects through pathogens and consumers (Abe et al., 2001; Nakashizuka, 1988). Consequently, once a canopy gap occurs, the gap may not be closed for an extended period, so that *S. kurilensis* would be able to keep taking advantage of spatial heterogeneity in light availability. Both experimental and theoretical works that emphasized the role of clonal integration in heterogeneous habitats mostly assumed that the spatial patterns of resource supply are fixed (e.g., Day et al., 2003; He et al., 2011; Roiloa et al., 2007) or change temporally independent of plant growth (Kun & Oborny, 2003; Mágori et al., 2003). The possibility of clonal species to manipulate local environments to be suitable for themselves by clonal expansion has received little attention (Chen, Li, Zhang, Zong, & Lei, 2015; Dickson et al., 2014) and deserve further studies for *S. kurilensis*.

In summary, our results suggest that the competitive interactions among genets and spatial expansion into closed canopies from higher‐light microsites likely underlie the proliferation of *S. kurilensis*. Light is clearly the most important resource limiting the growth and reproduction of plants growing in deciduous forest floors (Whigham, 2004). Many species (e.g., spring ephemerals) complete most of their growth and reproduction before canopy trees fully expand their leaves, while others persist in a vegetative state under closed canopies and begin to reproduce once gaps form (Kudoh et al., 1999; de Steven, 1989). This study suggests that *S. kurilensis* may have another strategy, namely the utilization of canopy gaps to proliferate throughout the forest understorey.

## CONFLICT OF INTEREST

None declared.

## AUTHOR CONTRIBUTIONS

A. Matsuo, HT, and A. Makita conceived the ideas and methodology; all authors collected the data; A. Matsuo and HT analyzed the data and led the writing of the manuscript.
