# Supplementary material for: Genet dynamics of a regenerating dwarf bamboo population across heterogeneous light environments in a temperate forest understorey
Source: Ecol Evol. 2018 Jan 8;8(3):1746–57. doi: 10.1002/ece3.3793 (PMC5792577; doi:10.1002/ece3.3793)
Supplement: Supplementary file 1 [file ECE3-8-1746-s001.docx]

**Supplementary Information**

**Fig. S1.** Light conditions, quantified as canopy openness, of six plots in 2005 and 2012. Canopy openness was estimated using hemispherical photographs, taken above the understory canopy of *Sasa kurilensis* (~3 m in height) at the center of each plot. The diagonal line is the 1:1 line.

**
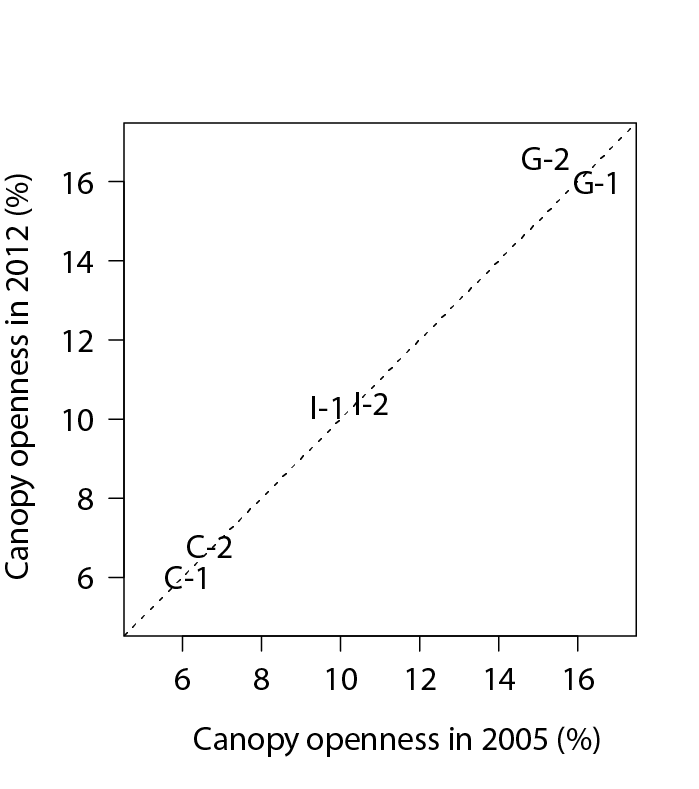
**

**Table S1.** Details on an additional microsatellite locus that had previously been developed for *Sasa* species.

| **Locus** | **Repeat motif** | **Primer sequences (5′–3ʹ)** | **Product length**  **(bp)** | **Reference** |
| --- | --- | --- | --- | --- |
| *ST57* | CT(GA)_3_AAGTGGATGCTATT | F: ACACACACACACAGAGAGAGAG  R: AATGTTGTCTGGCTCGAGGT | 218–236 | Sumiyoshi *et al.*, unpublished |

**Fig. S2.** Allometric relationship that explains the aboveground biomass of each culm, estimated by sampling 65 culms with a full range of sizes in the population. The coefficient of determination (*R*^2^) is 0.97. See text for details.

**Fig. S3.** Spatial distribution of culms and rhizomes for six plots in 2005 as revealed by the excavation of soil. Closed circles show the positions of culms, whereas lines and arrows show the rhizomes and their extending directions, respectively. Note that, although many genets already extended leptomorph rhizomes in canopy gaps, most of them have not yet produced culms on the leptomorph rhizomes (see Fig. S4 for discriminating genets).

**Fig. S4.** Spatial distribution of genets, identified using seven microsatellite loci, for six plots in 2005 and 2012. Numbers show the positions of culms and its genet identity in each plot. Note that the same numbers in different plots indicate different genets.

**Fig. S4.** Cont’d.
